# Sex hormones, aging and cardiometabolic syndrome

**DOI:** 10.1186/s13293-019-0246-6

**Published:** 2019-07-01

**Authors:** Jessica L. Faulkner, Eric J. Belin de Chantemèle

**Affiliations:** Department of Medicine (Cardiology), Vascular Biology Center, Medical College of Georgia at Augusta University, 1460 Laney Walker Blvd, Augusta, GA 30912 USA

**Keywords:** Sex differences, Sex hormones, Obesity, Diabetes, Hypertension, Endothelial function, Aging

## Abstract

It is well documented that the metabolic syndrome predisposes patients to increased cardiovascular risk. Emerging data indicates that cardiovascular risk conferred by metabolic syndrome is highly dependent on sex and sex hormone status throughout the lifetime. Both male and female sex hormones, as well as sex chromosomes themselves, contribute to the development of obesity and intervene in the control of insulin homeostasis and blood pressure. Furthermore, men and women develop age-associated cardiometabolic risk in a sex-specific fashion in association with changes in these sex hormonal levels. Therefore, the current notion of the metabolic syndrome as a sex-independent diagnosis is antiquated, and novel studies and clinical trials utilizing these known sex differences in the development of metabolic dysregulation and cardiometabolic risk are warranted.

## Background

The term “metabolic syndrome” is utilized today to refer to what has been formerly termed “insulin resistance syndrome,” “cardiometabolic syndrome,” or “syndrome X.” Metabolic syndrome encompasses a collection of risk factors related to dysregulated energy, insulin, and lipid homeostasis that collectively confer additive cardiovascular risk in men and women. The clinical use of metabolic syndrome is as a predictor of cardiovascular disease risk, and its diagnosis is associated with an increased likelihood of coronary artery disease, myocardial infarction, and stroke [1, 2]. The metabolic syndrome was first described in 1988 when it was noted that increased risk of coronary artery disease associated with insulin resistance commonly presents alongside other factors, notably dyslipidemia and hypertension, leading to a proposed collective “Syndrome X” of these risk factors for coronary artery disease [3]. The diagnosis of metabolic syndrome was then, and currently remains, a controversial topic due to discrepancies in criteria of inclusion and the individual risk afforded by those criteria. Emerging data of the contribution of obesity and central adiposity to insulin resistance risk evolved the diagnosis of metabolic syndrome to include central adiposity as a criteria, an important development as obesity continues to rise in prevalence. In 2009, a coalition of the National Heart, Lung, and Blood Institute, American Heart Association, World Heart Federation, International Atherosclerosis Society, and International Association for the Study of Obesity developed an overarching diagnostic criteria list for metabolic syndrome [4]. This consortium determined that at least 3 of the following diagnostic criteria must be met for diagnosis:

1. A waist circumference > 88 cm for women and > 102 cm inches for men (in the USA, variable criteria for other countries)^1^
2. Circulating triglyceride levels above 150 mg/dL (or drug treatment to lower triglyceride levels)
3. Circulating HDL-C cholesterol below 50 mg/dL for women and 40 mg/dL for men (or drug treatment to elevate HDL-C levels)
4. Blood pressure above 130/85 mmHg (or drug treatment to reduce elevated blood pressure)
5. Fasting blood glucose above 100 mg/dL (or drug treatment to reduce glycemia)

Importantly, sex is now emerging as a significant predictive factor in the development of cardiovascular diseases associated with metabolic dysregulation. As is apparent from the sex-specificity in some of these criteria, the contribution of these factors to cardiovascular risk in men and women is not ubiquitous. Notably, sex differences are consistently emerging in rates of obesity and insulin resistance, factors that are primary contributors to the other diagnostic criteria of metabolic syndrome. In addition, aging presents a unique challenge to the prediction of cardiometabolic risk in men and women as both sex hormone-dependent and sex hormone-independent effects play various roles in the development of aging-related cardiovascular diseases in men versus women. As the prevalence of metabolic syndrome is over 50% in both men and women over the age of 60, with a sharper recent increase in this prevalence in women [7], understanding individualized metabolic syndrome development based on sex is of immense clinical importance to population-wide cardiovascular risk.

The presence of either obesity or insulin resistance in men and women increases risk for coronary heart disease, vascular dysfunction, myocardial infarction, and stroke [1]. It is also well established that abdominal obesity and insulin resistance in and of themselves are risk factors for dyslipidemia [8–10], which encompasses two other criteria of the metabolic syndrome, specifically hypertriglyceridemia and low levels of high-density lipoprotein (HDL)-C. These two types of dyslipidemias are known risk factors for atherosclerosis, coronary artery disease, and other cardiovascular events [11, 12]. Triglyceride levels may be of particular importance to cardiovascular risk in obese men compared to women, as risk indices are increased in men compared to women for obesity [13] and insulin resistance [14]. However, despite that the prevalence of obesity and insulin resistance are not staving, improvements in dyslipidemia in both men and women have been dramatic population-wide in the last couple of decades, notably due to the increased prevalence of statins use [15]. In contrast, hypertension, the most prominent risk factor for cardiovascular events, is currently experiencing a rise in men and women, particularly young women [16, 17]. Alarmingly, rates of uncontrolled hypertension remain above 30% in both men and women, and may be rising in women, specifically [16, 18]. These data collectively indicate that the most pressing studies needed to alleviate the burden of cardiometabolic risk are those to reduce the incidence of obesity and insulin resistance and those to better control blood pressure in at-risk men and women. The focus of this review is to evaluate sex differences and the role of sex hormones and chromosomes on adiposity, insulin resistance, and hypertension that support the need for a redirection of treatment strategies that take into account that adiposity, insulin resistance, and hypertension are the predominant present-day risk factors for cardiometabolic events, and treatment strategies need to be sex-specific to improve outcomes in patients with metabolic syndrome.

## Main text

### Sex and adiposity

#### Obesity and visceral adiposity may be the primary factors underlying sex differences in prevalence of the metabolic syndrome

Epidemiological data indicates that increased waist circumference is the most prevalent criteria of the metabolic syndrome in men and in women [19]; however, metabolic syndrome as diagnosed by the current criteria is more prevalent in women than men [20]. This sex discrepancy is likely due to sex differences in the rates of obesity and central adiposity between men and women. It is important to note that waist circumference and obesity are not synonymous; however, waist circumference is strongly associated with a body mass index (BMI) ≥ 30 kg/m^2^ and is an additive, rather than exclusive, cardiovascular risk factor in obese patients [21, 22]. Several large-scale trials have demonstrated that rates of obesity are higher in women than men in the USA [23–27] and worldwide [28]. In addition, there is a disparity in the severity of obesity between men and women, as the prevalence of class III obesity (BMI > 40 kg/m^2^) is roughly 50% higher in women [26, 29, 30]. In association with rising rates of obesity, the prevalence of metabolic syndrome has risen more significantly in women of all age groups and races in the USA since the 1980s [31] indicating a cause-and-effect relationship of increasing BMI in women and prevalence of metabolic syndrome.

#### Visceral adiposity in men may be regulated primarily by testosterone levels

The sex-specific criteria for elevated waist circumference (> 102 cm for men vs. > 88 cm for women) make it slightly difficult to evaluate the effects of abdominal adiposity gram for gram between men and women. Mean waist circumference on average is higher in men compared to women (~ 95 cm in men vs. ~ 83 cm in women in the Tromso Study) [32], but more women are diagnosed with elevated waist circumference than men. This is an important discrepancy as waist to hip ratio in men is more highly clinically correlative with myocardial infarction than obesity as a general factor [33], indicating that waist circumference improvement, i.e., visceral adiposity, may be of particular importance in men to prevent cardiometabolic risk rather than weight loss alone.

A sex-specific predisposition for abdominal, or visceral, adiposity in men may be due to adipose-regulating effects of testosterone. Although aging men do not experience a menopause-like dramatic decrease in sex hormone levels as experienced by women, testosterone levels decline steadily with age in men [34]. These declining testosterone levels are associated with elevated visceral adiposity observed in aging men [35], which may play a role in increased cardiovascular risk in aging men. Testosterone therapy has been shown to increase muscle mass/decrease fat mass in older men [36, 37]; therefore, long-term therapy may improve metabolic health by improving lean/fat mass ratio. However, dosing and efficacy remain a challenge to these therapies as many trials are conducted in men with very low testosterone levels and also the observation that testosterone efficacy to limit adipose growth may slow with age [38]. In association, testosterone therapy may preserve the ability of adipocytes to conserve lipids with age indicating that aging-associated adiposity may be an evolutionary mechanism in men to preserve both lean and adipose mass [39], which presents a challenge to overcoming obesity-associated adiposity.

Association studies of testosterone levels in men and women offer a paradox for determining its true effects on visceral adiposity independent of sex hormones. Low serum testosterone is associated with reduced subcutaneous, and increased abdominal, adiposity in men [40–42], while high testosterone is associated with the same in women [43]. These data indicate that endogenous sex hormone- or chromosome-associated factors alter the effects of testosterone on adipogenesis in men and women. Whether testosterone acts to decrease abdominal adiposity independent of sex chromosomes is less clear, however. In women with androgen excess, a characteristic of polycystic ovarian syndrome (PCOS), estradiol + anti-androgen therapy is associated with reduced visceral adipose and higher lean mass in one study [44] and increased abdominal adiposity in another [45]. Therefore, further studies are needed to determine the true effect of anti-androgen therapy independent of estrogen on adipocyte deposition.

The mechanism(s) via which testosterone regulates adipose differentiation/deposition is likely an intra-adipose mechanism. Male rats supplemented with dehydroepiandrosterone (DHEA), a precursor of sex steroid hormones, developed decreases in visceral (epididymal) adiposity in vivo in association with reduced stromal vascular growth within the tissue, and further, DHEA inhibited murine adipocyte proliferation in vitro [46]. These experimental effects are likely derived from the androgen receptor in adipose tissue itself as male mice with both global [47] and adipose-specific [48] androgen receptor deficiency exhibit increased weight gain and visceral adipose accumulation. The effects of estrogen on adipose deposition may not be efficacious in the presence of testosterone in males, in contrast to females. Increasing aromatase activity via transgenic overexpression of the aromatase enzyme specifically in white adipose of male mice, which subsequently increases estrogen/estrogen receptor activation in white adipose, did not have an effect to alter fat or lean mass in male mice, although they do report an improvement in insulin sensitivity and adipose inflammation in this model [49]. In contrast, global aromatase knockout in male mice decreases lean mass and impairs insulin sensitivity in male mice, indicating that the effects of aromatase and its subsequent changes on adipose tissue function remain unclear.

The role of lean mass as a measure of improved cardiometabolic risk in many of these studies may be of particular importance as several reports have indicated that lean mass is increased by testosterone therapy in men, even in the absence of a change in fat mass [50, 51], a physiological change that may be attributable to the known function of testosterone to increase muscle pluripotent cell differentiation [52]. Therefore, the potential for testosterone therapies to limit visceral adiposity in men requires further study into the mechanisms via which the hormone regulates adipose deposition on a cellular level.

#### Female sex hormones promote subcutaneous adiposity

Women are more predisposed to obesity compared to men, and in association, women of all ages demonstrate a higher body fat percentage compared to men [53]. Prior to menopause, women predominantly deposit adipose in subcutaneous depots rather than viscerally [54]. Many studies, particularly those of women prior and post-menopause, demonstrate that female sex hormones strongly regulate adipose locale in women. Changes in circulating sex hormone levels associated with menopause are associated with distinct changes in adipose distribution patterns, reverting to visceral accumulation and increasing the likelihood of elevations of waist circumference [55–62]. In the Study of Women’s Health Across the Nation (SWAN) fat pattern study, it was demonstrated that low estrogen levels predicted visceral accumulation in women during and following menopause [43]. Female sex hormone effects on adipose deposition have also been reviewed in detail elsewhere [63].

In accordance with clinical studies, suppression of female sex hormones by ovariectomy in rodents increases gonadal (visceral), but not inguinal (subcutaneous) fat [64, 65]. Whether the effects of estradiol on adipose tissues is a direct effect has been postulated. Both visceral and subcutaneous adipose [66, 67] tissues express estrogen receptors. Estrogen receptor-α deletion specifically in adipocytes increases fat pad weights of both subcutaneous and visceral adipose depots in male and female mice, indicating that estrogen receptors promote, but do not necessarily regulate, adipose generation within the tissue itself [68, 69]. Furthermore, the fact that this was present in male and female mice indicates that intra-adipose effects of estrogen receptors to increase fat mass are not tied to sex-chromosomes, which indicates that sex hormone changes in menopause and their consequences to increase visceral adipose accumulation may involve other hormones, such as progesterone or testosterone. These studies also demonstrate that adipose dysfunction in response to adipose estrogen receptor deletion results in a systemic insulin-resistant phenotype, indicating a pronounced importance for these receptors for cardiometabolic health. The regulation of adipose function by adipose-specific estrogen receptors has been thoroughly reviewed elsewhere [70].

#### Sex hormones in obesity-associated and obesity-independent insulin resistance

Adiposity and obesity are heavily tied with insulin resistance, and increases in BMI on average correlate with higher fasting blood glucose across both sexes and at all ages. Similar to visceral adiposity, emerging evidence indicates that sex hormones play a significant role in insulin sensitivity both in lean and in obese men and women.

Studies indicate that elevated BMI produces a more pronounced insulin resistance phenotype in men than women [71]. Similar to visceral adiposity, hyperinsulinemia is also associated with low testosterone levels in men [40, 42]. Deficiency in androgen receptor signaling, both in male humans [72] and in rodents [47], conveys predisposition to insulin resistance. The insulin-sensitizing effects of testosterone have been attributed to androgen receptor activation in adipose and skeletal muscle promoting glucose uptake. Mice with global androgen receptor deficiency depict impaired glucose tolerance and lower phosphoinositide 3-kinase (PI3K) expression in skeletal muscles [47]. Aging in men is associated with progressive declines in skeletal muscle glucose uptake, which may be improved by testosterone therapy [73]. Although testosterone therapy may have benefit to improve glucose handling, whether hormone therapy will be more efficacious than currently available glucose-lowering pharmaceutical agents is currently unknown and warrants investigation. In a recent study, testosterone therapy did not improve insulin sensitivity as well as metformin [74]. Therefore, testosterone therapy for insulin resistance patients may be best aligned with concurrent glucose-lowering drugs for maximum efficacy. A more comprehensive review of the role of androgens on mechanisms of insulin sensitivity has been published elsewhere [75].

Studies of female sex hormone actions on insulin resistance have been primarily focused on estrogens. Estradiol has been shown to have direct actions to increase glucose uptake in skeletal muscle and adipocytes as well as anti-inflammatory and anti-oxidant effects to improve insulin receptor function indirectly, as has been reviewed extensively elsewhere [76, 77]. The most notable shift in insulin resistance in aging women occurs following menopause. Evidence is emerging that insulin resistance risk conferred from menopause in women may be alleviated by estradiol hormone replacement therapy, as has been reviewed [78]. Interestingly, in premenopausal women, phases of the menstrual cycle in which female sex hormones (estrogen and progesterone) are elevated are associated with an impairment in insulin sensitivity [79]. Therefore, the actions of progesterone may be antagonistic to those of estrogen on glucose uptake. This notion is supported by a study in which progesterone supplementation in female ovariectomized rats induced insulin resistance, while estrogen + progesterone combination did not [80]. These results may be attributed to the effects of progesterone to reduce glucose uptake in insulin responsive tissues, as progesterone supplementation has been shown to reduce glucose uptake markers in murine adipocytes [81]. With a distinct change in sex hormone status at menopause in women, the efficacy of glucose-lowering drugs in combination with hormone therapy (both oral contraceptive and post-menopausal supplement) is of paramount importance to determine optimum treatment strategies to improve glucose homeostasis in women across the lifespan.

#### Hypertension is a sex-specific predictor of cardiometabolic risk

Hypertension is a significant risk factor for cardiovascular disease mortality and a significant contributor to cardiovascular risk afforded by the metabolic syndrome [82, 83]. Risk of hypertension is increased by the prior presentation of any of the other criteria: dyslipidemia, increased waist circumference, and insulin resistance [10, 84]. Therefore, hypertension is likely more of a “consequence” rather than an originating cause of metabolic syndrome.

Importantly, the measure of hypertension in metabolic syndrome patients provides a significant inference of cardiometabolic risk in patients as clinical evidence indicates that hypertension may be the strongest single predictor of cardiovascular events [82, 83, 85]. Currently, the rising rates of hypertension in both men and women are closely correlated with, and clinically attributable to, increasing rates of obesity and insulin resistance [86, 87]. The metabolic syndrome criteria of 130/85 mmHg as a minimum for inclusion were considered “pre-hypertension” patients until very recently. The American Heart Association in a conjoined effort with the American College of Cardiology re-determined diagnostic criteria for hypertension, in contrast to previous diagnostic criteria of 140/90 mmHg [88]. These lower thresholds of blood pressure reflect the cardiovascular risk brought on by even slight increases in systolic and diastolic pressure, making the control of hypertension in patients with metabolic syndrome of utmost clinical importance. In addition, vascular endothelial dysfunction, which is closely associated with hypertension, confers additional risk of potentiating metabolic dysfunction via contributing to vascular inflammation, vasoconstriction, and impaired glucose clearance in tissues [89]. Therefore, the cardiometabolic risk conferred by hypertension may also serve to further impair insulin resistance in metabolic syndrome patients.

The contribution of adipose tissue to hypertension is sex-specific and associated with adipose distribution. It has been demonstrated that a given mass of visceral adipose tissue is associated with a greater increase in muscle sympathetic nerve activity, a measure of systemic sympathetic tone, than the same mass of adipose of a different depot [90–92]. In association, sympathetic activation has been shown to be a significant player in the development of hypertension in obese men, but not in young women, as reviewed in detail elsewhere [93]. The contribution of sex hormones to adipose deposition is evident in that visceral adiposity increases in postmenopausal women [58–62], which is concordantly associated with increased sympathetic nervous system tone in postmenopausal women [94]. In addition, sympathetic activation increases in both men [95] and women [96] with aging, implicating these measures gain importance as men and women age; however, mechanisms of cardiometabolic hypertension risk in younger women remain more elusive.

Overall, hypertension rates are higher in men than in women prior to ages associated with menopause; however, an alarming recent clinical trend indicates that hypertension prevalence is increasing in young women [17]. This increase is likely attributable to the strong association of obesity, insulin resistance, and hypertension in premenopausal women [97], the presentation of which negates cardiovascular protection that has been attributed to female sex hormones in young women [98, 99]. With the role of sympathetic tone in blood pressure control males and postmenopausal females well versed in the current literature, the question remains of mechanisms controlling blood pressure in young females with metabolic syndrome. Emerging data indicates the aldosterone-mineralocorticoid receptor axis may provide a mechanistic link between obesity and hypertension in young women. Clinical data indicates that mineralocorticoid receptor antagonism may be more efficacious for blood pressure reduction in women compared to men, albeit not in an age-adjusted population [100]. Furthermore, aldosterone levels are increased in closer association with BMI in women compared to men [101]. Recent studies from our group demonstrate that leptin increases adrenal aldosterone production, and increases serum aldosterone levels, in young obese female rodents which underlies the development of endothelial dysfunction and hypertension [102, 103]. A more detailed synopsis of this potential pathway for leptin-induced aldosterone-mediated hypertension risk in premenopausal women has been reviewed by the authors previously [104, 105].

#### Cardiometabolic risk may be increased by dissociation of sex chromosome complement to sex hormones

Sex chromosome complement to sex hormones may play a significant role in cardiometabolic risk. Experimentally, mouse models in which sex chromosome complement and sex hormones may be dissociated provide a promising avenue to begin to answer these questions. The 4-core genotype mouse model allows for the differentiation of effects of sex chromosomes from that of sex hormones by genetic modification to the Sry-male-determining region of the Y chromosome in males to create XY females and XX males. Independent of female or male sex hormones, mice with two X chromosomes developed higher body adiposity, but similar distribution patterns to females with intact sex hormone production, which was accompanied by increased insulin resistance and dyslipidemia [106]. In the same study, XY chromosome complement in mice was associated with elevated gonadal adipose weight [106], indicating a role for the non-Sry (testes-determining) region of the Y chromosome in the regulation of adipocyte mass and distribution. In addition, independent of alterations in adipose tissue mass, sex chromosome complement of XX is associated with a potentiation of angiotensin II-induced hypertension in the absence of female sex hormones [107]. This study indicates that changes in female sex hormones in women may have a more pronounced effect directly on blood pressure control in women, a factor to be considered in obese women with hypertension. Therefore, both sex chromosome complement and hormone status play a role in cardiometabolic consequences of adiposity, which strengthens the need for data-inspired therapeutic regimens based on both of these aspects of sex in metabolic syndrome patients.

#### Efficacy of sex hormone therapy for cardiometabolic risk prevention is dependent on dosing factors: evidence from postmenopausal women and transgender therapies

It would be convenient to say that hormone supplement therapy to aging men and women with reduced testosterone or estradiol levels is the key to reducing their likelihood of metabolic syndrome-associated cardiovascular events. This complication is apparent in data from the Women’s Health Initiative, in which equine estrogens were administered alone or in combination with medroxyprogesterone acetate to over 100,000 postmenopausal women. The results from this study, which overall determined an increased risk for thromboembolytic events and coronary heart disease in response to hormone therapy, resulted in a large-scale clinical brake on postmenopausal hormone replacement therapy [108, 109]. However, since the release of these conclusions, a number of studies have emerged demonstrating a potential benefit for controlled postmenopausal estrogen therapies, with an added focus on dosing, formulation, and menopausal status at initial administration, which has been reviewed elsewhere [110].

Studies of hormone therapy in transgender individuals offer a cohort in which to determine a cause and effect relationship of sex hormones to cardiometabolic risk as the endogenous sex hormones are usually suppressed simultaneously to hormone supplementation. The potential benefit to these studies is primarily the ability to render effects attributable to the hormones themselves independent of sex chromosomal effects in a human population. However, it is important to note that dosing and hormone regimens as well as adherence is highly variable in these studies and the results are therefore difficult to extrapolate in certain studies, in addition to the confounding factor of the developmental exposure to the patients’ endogenous sex hormones for variable amounts of time. However, these patients do shed some light into the potential effects that hormone therapies have on the characteristics of metabolic syndrome.

Studies have shown that, overall, risk of myocardial infarction is increased in transgender-identifying individuals, both male and female [111], and that gender reassignment surgery and hormone therapies are associated with a 2+-fold increase in odds risk for death by cardiovascular diseases [112]. Data in transgender men to women transition indicate that estrogen therapy combined with an antiandrogen conveys an increased risk of cardiovascular disease-related death [113]. However, other data on estrogen therapy and cardiovascular risk do not convey similar associations [114], and in addition, these studies do not necessarily account for other cardiovascular risk factors that may be more prevalent among transgender individuals, such as a higher prevalence of smoking [115]. However, with the known cardiovascular effects of sex hormones and the data in mice conferred by genomic sex chromosome complement models, it is likely that estrogen and testosterone may confer cardiometabolic protection only when accompanied by a certain sex chromosome complement. However, dosing is notably inconsistent throughout most studies of transgender hormone therapy, rendering a generalization of the role of hormone therapies in cardiovascular risk difficult to determine at present. A comprehensive review of studies of hormone therapy and associated cardiovascular factors has been published previously [114]. Collectively, the presentation of various outcomes of cardiometabolic risk in these patients undergoing hormone replacement therapies highlights the importance of continued study into efficacious dosing and formulations of hormone therapies both in cisgender and transgender patients.

## Conclusion

Sex-discrepant prevalence of adiposity and insulin resistance render differences in cardiometabolic risk between men and women. The promise of therapies for adiposity and insulin resistance may include sex hormone supplements; however, many strides are needed to determine the appropriate treatment dosages and outcomes and consideration of chromosome complement effects. Future trials, some of which are currently ongoing, will continue to provide evidence for the appropriate determination of the effects of individual sex hormones on metabolic function in men and women at all ages [116]. In addition, with the advent of statins for the control of dyslipidemia, prevention of hypertension risk is a crucial missing link to improvement of cardiometabolic risk.

## Data Availability

Not applicable

## Abbreviations

BMI: Body mass index
DHEA: Dehydroepiandrosterone
HDL: High-density lipolipids
PCOS: Polycystic ovarian syndrome
PI3K: Phosphoinositide 3-kinase

## Footnotes

1 Importantly, it has been suggested that ethnicity-specific criteria for waist circumference be established to optimally predict cardiometabolic risk [5, 6], although this is not currently included in the metabolic syndrome diagnosis.
